# Benchmarking 16S rRNA Gene-Based Approaches to Bacterial Taxonomy Assignment Based on Amplicon Sequencing With Illumina and Oxford Nanopore

**DOI:** 10.1155/ijm/7563096

**Published:** 2025-08-13

**Authors:** Carmen Hoffbeck, Danielle M. R. L. Middleton, Nicola J. Nelson, Michael W. Taylor

**Affiliations:** ^1^School of Biological Sciences, University of Auckland, Auckland, New Zealand; ^2^Manaaki Whenua – Landcare Research, Lincoln, New Zealand; ^3^School of Biological Sciences, Victoria University of Wellington, Wellington, New Zealand

## Abstract

Research investigating the microbial community of an ecosystem or animal can involve a range of methodologies, including sequencing technology, bioinformatic software and taxonomy database. Researchers may utilise short-read sequencing on Illumina MiSeq or long-read sequencing on platforms like Oxford Nanopore to obtain different research outcomes, for example, enhanced identification of microbes at species or strain level with Nanopore. However, replicability across these techniques is not well studied, while the technique used to process reads into microbial taxa may also result in different taxonomy assignments. In this study, we analyse an existing, real-world dataset which had low genus-level identification with Illumina sequencing and analysis with the SILVA database and compare sequencing with Nanopore on the same samples. We pair this with multiple bioinformatic approaches and taxonomy databases for each sequencing technique to compare phylum- and genus-level assignments and use mock communities to identify which combination of sequencing technique, bioinformatic approach and taxonomy database provides the most accurate taxonomy. We found that Nanopore reads processed with either utilised bioinformatic approach or taxonomy database provided higher accuracy in the assignment of a mock community than any technique combination with Illumina. We also found that the Top 10 genera assigned to a real-world database were substantially different across technique combinations and varied more by taxonomy database than either bioinformatic approach or sequencing technology.

## 1. Introduction

The microbiota of an organism or environment refers to the collection of all microorganisms living therein, potentially comprising thousands of microbial species. These microbial communities have increasingly become the focus of research into human health [1–3], soil productivity [4–6] and endangered species conservation [7], among many other topics. To identify the microbes present in a community, researchers typically employ genetic extraction and sequencing to obtain the microbial DNA or RNA and utilise bioinformatic approaches to assign taxonomy to the sequenced genetic material. Correctly identifying the microbes present in a sample can assist in disease diagnosis [8, 9], predict host physiology and development [10–12] and shine a light on other important determinants of host and ecosystem health. The identification of microbes must therefore be accurate and provide sufficient resolution (e.g., to microbial genus or species level) to correctly interpret any differences among samples or between study systems.

In the last decade, second-generation Illumina sequencing (San Diego, California) has become the most commonly used tool for sequencing microbial DNA. Microbiota studies typically target an amplicon, a PCR-amplified region of a gene of interest, producing short reads for highly variable regions such as the 16S rRNA gene in bacteria and archaea [13] and the ITS region in fungi [14]. Illumina sequencing provides low error rates on bases from amplicons, but often low taxonomic resolution (where sequences are assigned to ‘unknown' genus or species) due to the relatively short genetic region available for mapping [15]. Bioinformatic approaches for analysing Illumina data often do not assign taxonomy at the species level, and even at genus level, some organisms are too closely related to be differentiated by partial 16S rRNA gene sequencing [16]. As many researchers analyse only a portion of a gene, for example, the V3–V4 regions of 16S rRNA genes, much bioinformatic power to assign microbial taxonomy is lost, particularly for databases such as those maintained by NCBI that match the gene fragment against every submission, including whole genomes [17, 18]. This also constrains comparisons across studies where different variable regions were used for sequencing [15, 19]. Though Illumina sequencing provides adequate coverage for a number of research applications, many research questions could be more fully addressed with greater taxonomic resolution.

Recently, third-generation sequencing technology such as Oxford Nanopore (Oxford, United Kingdom) has provided longer read sequencing for lower up-front costs than with similar sample volumes on Illumina [20]. This technology allows for full-length sequencing of discriminatory markers such as 16S rRNA genes, which may provide greater resolution at the genus or species level for some microbes [21–24]. Particularly, longer read sequencing on Nanopore and PacBio has been useful as a supplement to short-read sequencing where lower taxonomic ranks cannot be reliably assigned [24] and in clinical settings where species- or strain-level identification is crucial [8, 9, 25]. Though Nanopore provides longer sequence reads, historically, this technology has generated more errors [26], which can lead to lower resolution. However, this drawback is diminishing, with the 16S Barcoding Kit SQK-RAB204 for Nanopore showing fewer errors than previous kits [15]. To evaluate if Nanopore is as effective as Illumina in some cases, multiple studies have compared the two sequencing techniques in ecosystems including the human gut [15, 27–29], soil and water [30–32] and mouse models [33]. Multiple studies reported little difference between Illumina and Nanopore in terms of assignment at genus level or higher [25, 32, 34], while others found that Nanopore performed better at identifying to species or genus [28, 33, 35, 36]. As noted in previous studies, the most effective comparisons will include a combination of mock communities and real bacterial communities, replicate analysis with the same samples in different bioinformatic approaches and evaluate community composition and diversity holistically [15].

The main dataset used in this study originates from an animal microbiome study of the tuatara, a reptile endemic to New Zealand with relevance to ecology and conservation. When analysed with Illumina 16S rRNA gene (V3–V4 regions) sequencing, many of the bacteria could not be identified at the genus level, limiting understanding of what these bacteria are and how they might be contributing to their host species [37]. Here, we investigate the real-world microbial dataset for this unique host species and a mock dataset using two sequencing technologies (short-read Illumina sequencing and long-read Nanopore sequencing), four bioinformatic approaches (DADA2 in R and with QIIME2, EPI2ME and Emu) and four taxonomic databases (SILVA, NCBI, GreenGenes2 and GTDB). This work represents a comparison between technique combinations on both a real-world dataset and mock communities of known composition, with identical DNA extracts put through both sequencing systems. We examine both the taxonomic output of each technique and the diversity of the resulting community. We also compare commonly utilised bioinformatic approaches and taxonomy databases for each output to determine the replicability of alpha and beta diversity findings across techniques for this real-world dataset.

## 2. Materials and Methods

### 2.1. DNA Extraction and Amplification

DNA was obtained from cloacal swabs of 174 tuatara, an endemic reptile of New Zealand (New Zealand Department of Conservation permit #50568-FAU), which were stored in RNA*later* at −20°C (~4°C for some samples, when necessary) until extraction. Further sampling details and methodology can be found in [37], with Illumina sequences uploaded to NCBI at PRJNA1008362. DNA was then extracted using a QIAamp Fast DNA Stool Kit (Qiagen, Valencia, California), as recommended by the manufacturer for bacterial DNA collection, and this extract was stored at −20°C until amplification. Three extraction blanks (replicates where no DNA was added) were also extracted using the same protocol and included in sequencing.

For Illumina sequencing, the V3–V4 region of the 16S rRNA gene was PCR-amplified using the 341F-785R primer pair [38] and KAPA 3G kit (Kapa Biosystems, Inc., Wilmington, Massachusetts) for tuatara samples and a Zymo mock community (ZymoBIOMICS Microbial Community Standard, #D6300; Zymo Research, Irvine, California). Thermal cycling conditions were as follows: initial denaturation at 95°C for 3 min, then 35 cycles of denaturation at 95°C for 20 s, annealing at 57°C for 15 s and extension at 72°C for 30s, followed by a final extension at 72°C for 1 min [37]. Seven samples were also amplified with 30 cycles for comparison. The presence of a correct-sized amplicon was confirmed using 1% agarose gel electrophoresis, and DNA quantity was measured using an EnSpire Multimode Plate Reader. Following amplification, 10 *μ*L of PCR product from each sample was purified using the Zymo ZR-96 DNA Clean-up Kit and sequenced using Illumina MiSeq (2 × 300 bp chemistry) by Auckland Genomics Ltd.

For Oxford Nanopore sequencing, the near-entire 16S rRNA gene was amplified from tuatara samples and a Zymo mock community (ZymoBIOMICS Microbial Community DNA Standard, #D6305) using the ONT27F-ONT1492R primer pair [39] and LongAmp *Taq* 2X Master Mix. The following thermal cycling conditions were used: initial denaturation at 94°C for 30 s, then 30 cycles of denaturation at 94°C for 30 s, annealing at 55°C for 30 s and extension at 65°C for 30 s, followed by a final extension at 65°C for 10 min. Following amplification, 10 *μ*L PCR product from each sample was purified using the Zymo ZR-96 DNA Clean-up Kit and sequenced on Oxford Nanopore GridION by Auckland Genomics Ltd using the 16S Barcoding Kit SQK-RAB204. Nanopore sequences have been uploaded at PRJNA108242.

### 2.2. Short-Read Bioinformatic Processing

**Figure 1:**
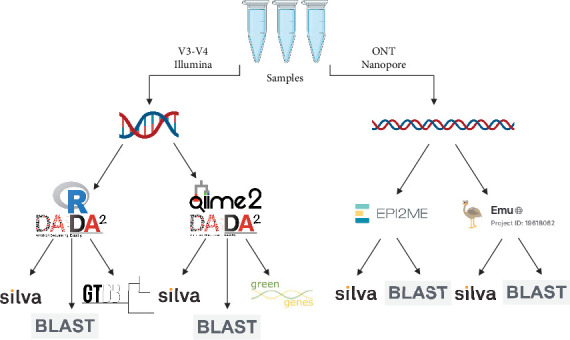
Workflow for comparative processing of 16S rRNA gene amplicon reads from Illumina and full-length 16S rRNA gene reads from Nanopore.

All reads were processed on the New Zealand eScience Infrastructure (NeSI) computing cluster. Illumina-produced reads underwent two different series of steps (hereafter called ‘bioinformatic approach') to process raw sequence reads into ASVs, namely DADA2 in R [40] and DADA2 with QIIME2 [41]. After reads were processed into ASVs with each of these bioinformatic approaches, three taxonomy databases were used to assign taxonomy to each approach (Figure 1). For DADA2 in R, these were SILVA-138 [42], GTDB-r207 [43] and the NCBI gene sequence database using command line BLAST [44]. For DADA2 with QIIME2, these were SILVA-138, Greengenes2 [45] and the NCBI gene sequence database using BLAST.

For both approaches, Illumina adaptors and primers were first removed from each sample using Trimmomatic with standard settings [46]. Sequence reads were then processed into ASVs using the DADA2 algorithm, with sequences trimmed and filtered for quality at 280 forward bp and 200 reverse bp and merged. Taxonomy was assigned as far as the genus level for the remaining samples using the SILVA-138 database or the GTDB database. Illumina sequence reads were also processed using DADA2 with QIIME2 with standard settings, again filtering at 280 forward bp and 200 reverse bp (Figure 1). Note that due to recent changes in bacterial taxonomy at the phylum level [47], some databases utilise new phylum designations while others retain the previous designations (e.g., SILVA uses the phylum designation Firmicutes, while NCBI uses Bacillota). These have been consolidated under the same colours on figures wherever possible. For ASVs produced with both DADA2 in R and through DADA2 with QIIME2, a fasta file of all ASVs was generated and used as input to command-line BLAST, where these ASVs were assigned taxonomy using the NCBI gene sequence database. This processing resulted in three ASV count tables and taxonomy tables from DADA2 in R and three ASV count tables and taxonomy tables from DADA2 with QIIME2.

### 2.3. Long-Read Bioinformatic Processing

Nanopore-produced reads were analysed with two bioinformatic approaches, EPI2ME (Oxford Nanopore) and Emu [22]. Reads which were rebasecalled using ‘super high accuracy basecalling' after the Nanopore run were used for downstream analysis and were subjected to trimming to 1500 bases and filtering to Quality Score 10 with NanoFilt [48]. These filtered reads were then processed using EPI2ME with the built-in SILVA-138 database and the built-in NCBI-16S-18S-ITS database and processed with Emu with the built-in SILVA-138 database and a curated version of the NCBI database combined with RDP v11.5 (as recommended in [22]). Though EPI2ME and Emu do not produce ASVs per se, they produce species- or strain-level assignments which can then be counted in each sample, allowing for the compilation of a count table for these assignments similar to an ASV table (though note that this process with Nanopore creates counts of species-level assignments rather than ASVs). This processing produced four count tables and four taxonomy tables. A step-by-step workflow of all bioinformatic approaches and taxonomy assignments is included in the Supporting Information.

### 2.4. Data Analysis and Visualisation

All data analysis and figure production occurred in R (Ver 4.2.2). Count tables and taxonomy tables from each technique combination were merged into individual phyloseq objects ([49]; Ver 1.42.0). The distance between the known mock community and the community profile produced by each technique combination was calculated using the Jaccard distance. Bacterial community alpha diversity was calculated for groups of samples within each bioinformatic approach (not from the merged phyloseq object) with the plot_richness function in phyloseq using the observed diversity metric, and significant differences between groups were determined by the Wilcoxon test. This allowed comparison of unique ASVs from DADA2 in R and with QIIME2 and of species-level identifications (number of taxonomic units, or ‘NTUs') from EPI2ME and Emu, rather than by the number of unique genera. In addition to these analyses, ASVs produced from DADA2 in R and with QIIME2 were mapped to Nanopore reads using BBMap ([50]; Ver 38.18), requiring first a 100% identity match and then a 95% identity match between Illumina-derived sequences and Nanopore-derived sequences.

## 3. Results

### 3.1. Taxonomy Assigned to a Mock Community of Known Composition

We first compared the taxonomic profiles from a community of known taxonomic composition, namely, the Zymo mock DNA communities sequenced with Illumina and Nanopore. The Illumina community consisted of 12% each of DNA from *Pseudomonas aeruginosa*, *Escherichia coli*, *Salmonella enterica*, *Limosilactobacillus fermentum*, *Enterococcus faecalis*, *Staphylococcus aureus*, *Listeria monocytogenes* and *Bacillus subtilis* (with the remaining 4% comprised of the fungi *Saccharomyces cerevisiae* and *Cryptococcus neoformans*, which were filtered from this bacterial analysis). The Nanopore community was made up of 4% *Pseudomonas aeruginosa*, 10% *Escherichia coli*, 10% *Salmonella enterica*, 18% *Lactobacillus fermentum*, 10% *Enterococcus faecalis*, 16% *Staphylococcus aureus*, 14% *Listeria monocytogenes* and 17% *Bacillus subtilis*.

**Figure 2:**
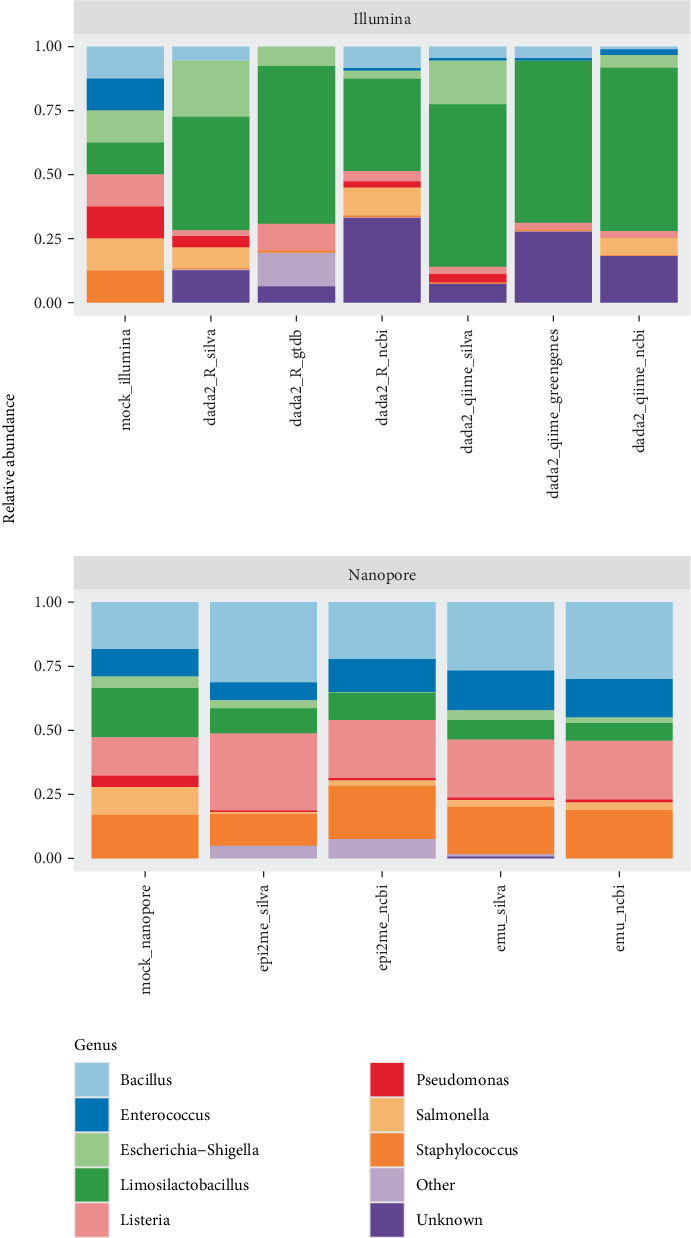
Zymo mock communities sequenced with Illumina and Nanopore. Expected communities are compared with the output from each approach and taxonomy database.

Among Illumina-derived sequences, those assigned using DADA2 with QIIME2/SILVA and DADA2 in R/GTDB provided fewer unknown bacteria overall (~10%). The most accurate communities were assigned using DADA2 in R/SILVA (Jaccard distance of 0.68), which correctly identified *Salmonella enterica*, *Bacillus subtilis*, *Escherichia coli* and *Pseudomonas aeruginosa* in similar proportions to the true community, though this combination led to an underestimation of *Staphylococcus aureus* and *Listeria monocytogenes*, a complete misidentification of *Enterococcus faecalis*, and overrepresented *Limosilactobacillus fermentum* (Figure 2). None of the approach/taxonomy databases used for Illumina reads achieved a Jaccard distance of less than 0.5 (Table 1), and all technique combinations failed to identify some of the mock community members at the genus level.

For Nanopore-derived sequences, taxonomy assigned with EPI2ME largely resembled the mock community when assigned both with NCBI (Jaccard distance of 0.500) and SILVA (Jaccard distance of 0.400). All eight bacterial genera in the mock community were identified, though some in much smaller proportions than expected, particularly *Salmonella*. Both databases with EPI2ME more accurately reflected the inputted mock community than any bioinformatic approach with Illumina and were able to identify all bacteria in the sample to the genus level (Figure 2). Mock community profiles assigned by Emu largely resembled those assigned by EPI2ME, with consistency across the two taxonomy databases used. All bacterial genera were identified in the mock community with Emu (Figure 2), and reads processed with Emu and SILVA had the lowest Jaccard distance to the mock community (0.386, Table 1).

### 3.2. Taxonomy Assigned to a Real-World Bacterial Community

**Figure 3:**
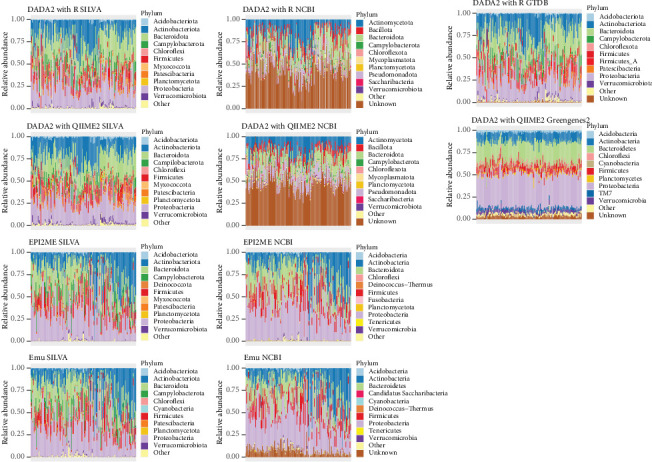
The 10 most abundant bacterial phyla, other phyla and unknown phyla assigned by each technique combination (Row 1: DADA2 in R, Row 2: DADA2 with QIIME2, Row 3: EPI2ME and Row 4: Emu) for the tuatara 16S rRNA gene dataset.

We next compared the phyla identified in the tuatara samples using each technique combination. The Top 4 phyla were consistently identified across techniques as Actinobacteriota, Bacteroidota, Firmicutes and Proteobacteria, though the relative proportions of these varied overall between techniques and across the same sample analysed with different techniques (Figure 3). Illumina samples with taxonomy assigned with NCBI had a far higher number of sequence reads which could not be assigned at the phylum level (~50%) compared with other taxonomy databases. By contrast, there were fewer unidentified phyla when analysing Nanopore samples with Emu (~10%) and no unidentified phyla when analysed with EPI2ME (Figure 3).

**Figure 4:**
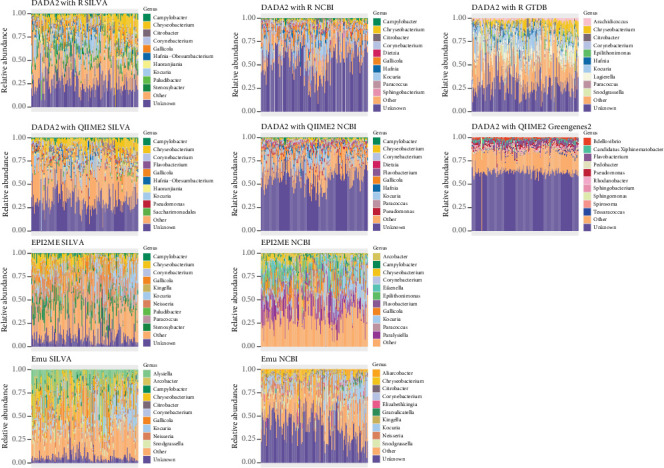
The 10 most abundant bacterial genera, other genera and unknown genera assigned by each technique combination (Row 1: DADA2 in R, Row 2: DADA2 with QIIME2, Row 3: EPI2ME and Row 4: Emu).

The bacterial genera with the highest relative abundance in the tuatara samples varied more between techniques, particularly between Illumina and Nanopore datasets (Figure 4). The only bacterial genera which were identified in the Top 10 of multiple techniques were *Campylobacter*, *Chryseobacterium*, *Corynebacterium*, *Flavobacterium*, *Gallicola*, *Kocuria*, *Pseudomonas* and *Sphingobacterium*, with *Chryseobacterium* identified in all technique combinations except DADA2 with QIIME2/Greengenes2. Among the Top 10 genera assigned with DADA2 with QIIME2/Greengenes2 were five genera which did not appear in any other combinations. The genus *Neisseria* appeared as one of the top genera among long-read samples processed with both EPI2ME and Emu but was never assigned in short-read samples processed with any technique. The percentage of unknown bacterial genera ranged widely between techniques, with ~60% with DADA2 in R/NCBI, DADA2 with QIIME2/NCBI and DADA2 with QIIME2/Greengenes2; ~30% with Emu/NCBI, DADA2 in R/SILVA, DADA2 in R/GTDB and DADA2 with QIIME2/SILVA; ~10% with EPI2ME/SILVA and Emu/SILVA and no bacteria unidentified at genus level with EPI2ME/NCBI (Figure 4).

When requiring a 100% match between Illumina ASVs and Nanopore sequences, only 0.6% of DADA2/R ASVs and 0.3% of DADA2/QIIME2 ASVs successfully mapped (Table S3). However, when allowing a 95% match, 78.8% of DADA2/R ASVs and 76.8% of DADA2/QIIME2 reads mapped to the Nanopore dataset.

### 3.3. Bacterial Alpha Diversity of Samples Produced Through Each Technique

**Figure 5:**
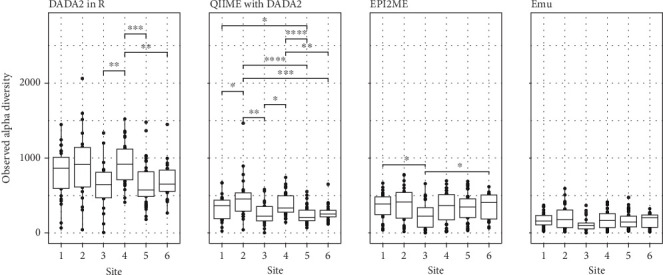
Observed bacterial community alpha diversity (richness) between field collection sites, compared between count tables generated by DADA2 in R, DADA2 with QIIME2, EPI2ME and Emu. ⁣^∗^*p* < 0.05, ⁣^∗∗^*p* < 0.01, ⁣^∗∗∗^*p* < 0.001 and ⁣^∗∗∗∗^*p* < 0.0001.

Finally, we examined any differences in observed alpha diversity across the techniques. We found differences in overall alpha diversity (here, ASV or NTU count) between DADA2 in R, DADA2 with QIIME2, EPI2ME and Emu, as well as different outcomes to hypotheses about alpha diversity in the data. These data were collected from six sites, ranging from north (Site 1) to south (Site 6) [37]. When analysed with DADA2 in R, significant differences were found between Site 4 and Sites 3, 5 and 6 (Figure 5). However, when analysed with DADA2 with QIIME2, additional significance was identified between Site 1 and Sites 2 and 5 and between Site 2 and Sites 3, 5 and 6. When analysed with EPI2ME, only Site 3 and Sites 1 and 6 were significantly different, and when analysed with Emu, significant differences were only found between Sites 1 and 3. Overall alpha diversity also varied between techniques, with Illumina samples displaying overall higher diversity than Nanopore samples (as expected due to processing differences, see the Discussion section). The DADA2 in R sample with the highest alpha diversity contained over 2000 ASVs, while no samples processed with EPI2ME contained more than 1000 NTUs and no samples processed with Emu contained more than 500 NTUs (Figure 5).

## 4. Discussion

This study utilised two sequencing platforms, four bioinformatic approaches and four taxonomy databases to compare genus-level taxonomy for both mock communities and a real animal microbiome dataset collected from 174 individual hosts. Using these data, we explored (1) how accurately each technique combination assigned taxonomy to a known mock community, (2) how taxonomy was assigned to a real dataset at phylum and genus level and (3) whether alpha diversity varied between technique combinations, and if variation introduced by technique could alter biological interpretations from a given dataset. Our findings suggest that choices in technique combination could substantially influence both the reported dominant bacterial genera and trends in alpha diversity, particularly in database selection.

### 4.1. Taxonomy of Samples Varied With Technique Combination

Despite identical DNA extracts being processed through all technique combinations, there were differences between the resulting bacterial community profiles. This mirrors findings in previous studies, where sequencing with 454 pyrosequencing and Illumina sequencing has been shown to introduce less variation than the 16S rRNA gene region sequenced [51]. These differences are a known pitfall of 16S rRNA gene analyses. A combination of bias in which microbes are submitted to and included in a given database, as well as errors in annotation within databases [52], can influence the nearest taxonomy match to which a gene sequence is assigned. For example, common human pathogens are represented with great detail in most databases, while environmental microbes are often underrepresented (*Pseudomonas* has 55,533 available genomes published in NCBI while *Gallicola* has four at the time of writing). The SILVA database is highly curated for alignment with short amplicons [42], while NCBI contains a vast array of sequences of varying lengths and annotation quality. Due to differences like these, utilising different taxonomy databases is known to introduce bias into 16S rRNA gene analyses [53]. Choice of sequencing platform is also known to introduce a degree of bias which might influence results across platforms [54], particularly when different gene regions have been sequenced. The mismatch between primers and targeted binding sites is higher for some bacterial taxa [38], introducing more bias when comparing between products amplified with different primer pairs. The bioinformatic approaches used to process raw reads also introduce a degree of variation, though comparisons have previously highlighted that differences are largely confined to relative abundance and alpha diversity rather than to absolute assignments which would change the biological interpretation [51, 55].

### 4.2. Technique Combination Substantially Influences Interpretation of Tuatara Microbiome Data

The high degree of variation in taxonomy assignments introduced by different technique combinations led to only marginal differences in the Top 10 bacterial phyla (Figure 3), but variable assignment to the Top 10 bacterial genera (Figure 4). Though a few genera were repeatedly counted in the Top 10 across multiple technique combinations (*Campylobacter*, *Chryseobacterium*, *Corynebacterium*, *Flavobacterium*, *Gallicola*, *Kocuria*, *Pseudomonas* and *Sphingobacterium*), none of these were listed in the Top 10 genera resulting from every combination. This is likely due in part to the large number of ‘unknown' genus-level assignments produced from Illumina-derived sequences, which in some cases accounted for the majority of assignments (Figure 4). Reporting of the dominant bacteria in this host and interpretation of the similarity of the tuatara community to that of other hosts would be substantially influenced by the technique combination, particularly the taxonomy database.

Ultimately, there is no way to identify the most ‘correct' interpretation of the real-world dataset used here. The unknown genera assigned using short reads with all approaches and databases could in principle represent truly undescribed bacterial genera, described bacteria absent from the utilised databases, or nonbacterial reads amplified due to primer misalignment [56, 57], and the true identity of these genera cannot be established by comparison. Conversely, identified bacteria may represent true matches or merely a match to a close relative in the database, particularly in cases where the host likely contains poorly described bacteria. Though genus-level assignment in the real-world dataset was variable across technique combinations, the assignments for the mock community profile were more consistent. The genus *Limosilactobacillus* (formerly *Lactobacillus*) was overrepresented in all Illumina-derived profiles by a large margin, in all cases reported as the most dominant community member (Figure 2). Conversely, the genus *Salmonella* was frequently underrepresented across technique combinations regardless of sequencing platform or approach and was completely absent from some reported profiles (such as DADA2 in R/GTDB and DADA2 with QIIME2/SILVA). *Salmonella* can be misidentified as other, closely related members of the family Enterobacteriaceae [58–60], though it is routinely accounted for in mock communities and is well documented in human microbiome studies. The primer pair 341F-785R has been shown to broadly amplify bacterial phyla [38] and *Salmonella* in particular [58], so the lack of identification with Illumina sequencing here warrants deeper investigation.

Reported bacterial alpha diversity was impacted by the bioinformatic approach utilised, with particularly stark contrasts in differences among sites between Illumina- and Nanopore-derived reads (Figure 5). Overall alpha diversity was highest for samples processed using DADA2 in R, with one sample having an observed alpha diversity of over 2000 ASVs (Figure 5). By comparison, the most diverse Illumina-DADA2 with QIIME2 sample contained ~1500 ASVs, while no sample sequenced on Nanopore contained more than 1000 NTUs. Although DADA2 with QIIME2-processed samples had lower overall alpha diversity, the difference between biologically relevant groups was more significant. When processed with DADA2 with QIIME2, Site 2 differed significantly from Sites 1, 3, 5 and 6, but when processed with DADA2 in R, EPI2ME and Emu, the alpha diversity of Site 2 was not significantly different from that of any other site. Compared with eight significant contrasts when reported from DADA2 with QIIME2-processed samples, Emu-processed samples contained only one significant contrast (Figure 5). Previous research has shown that DADA2 diverges marginally less than QIIME2 from expected alpha diversity [61], perhaps accounting for differences between DADA2 in R- and DADA2 with QIIME2-processed samples here.

### 4.3. Further Considerations

Differences in laboratory preparation potentially contributed to differences between reported community profiles derived from Illumina and Nanopore. Samples sequenced on Illumina were at a higher concentration than Nanopore samples before sequencing, likely due to shearing of the longer reads during the spin-column–based PCR clean-up stage [62]. Additionally, following manufacturer recommendations for amplification with ONT27-ONT1492 (Oxford Nanopore) and 314F-785R (with the KAPA 3G kit), primer pairs led to differing cycle numbers (30 for Nanopore and 35 for KAPA 3G). The additional cycles with Illumina potentially introduce further bias in both alpha and beta diversity [51, 55]. Input concentration, cycle number and the poor affinity of the 27F-1492R binding sites for some bacterial taxa [38, 63] may all contribute to variation when comparing Illumina and Nanopore reads. However, when considering identical extracts which were amplified with 30 or 35 cycles and both sequenced on Illumina, there was no significant difference in bacterial diversity (Figure S2). Likewise, the mean read number from files produced from Illumina and Nanopore was not substantially different (on average, Nanopore files had 92,915 reads, and Illumina files had 84,709; Table S2). Despite this, when examining alpha diversity via rarefaction curves, it appears that while Illumina sampling depth was sufficient to capture the full alpha diversity of samples, Nanopore samples processed with EPI2ME (though not Emu) have not yet reached an asymptote, indicating that more depth may be necessary to fully elucidate alpha diversity from these EPI2ME-processed samples (Figure S1).

Importantly, the process of assigning ‘ASVs' differs between short and long-read sequences. With Illumina sequences processed using DADA2 in R or with DADA2 with QIIME2, the raw sequences are denoised to remove duplicate sequences, and the software trains itself against the dataset of reads to determine the acceptable error rate. After this, ASVs are determined as sequences which differ from one another by more than the error rate, sometimes by as little as a single nucleotide [64]. After reads have been error-corrected and ASVs are assigned, taxonomy is assigned. With Nanopore sequences, software such as Emu assigns taxonomy directly to the longer reads, and error correction is performed afterwards based on total mapping counts in the sample [22]. The reverse process of assigning ASVs and taxonomy possibly overrepresents ASVs in Illumina samples, as many ASVs will represent variants of the same bacterial species, and possibly underrepresents ‘ASVs' in Nanopore samples as different strains or species may be assigned to the same higher classification [26]. We thus recommend caution when comparing actual alpha diversity estimates between Illumina and Nanopore technologies and instead emphasise the marked differences in biological interpretations of alpha diversity (i.e., which samples differed significantly) between the two platforms. Despite all the above sources of variation between Illumina- and Nanopore-derived reads, the sequencing platform nonetheless played a smaller part in variation than did the taxonomy database.

Determining how specific NTUs have been assigned across different approaches is not currently possible due to the proprietary nature of EPI2ME, but it would be useful to determine which sequences are being commonly misassigned or assigned to unknown genera. An ASV or NTU assigned to an ‘unknown' genus could represent three possibilities: (1) that the sequence is not present in the chosen database, (2) that the sequence is in the database but has a 16S rRNA region that does not allow classification at genus level or (3) that the sequence is present in the database but belongs to an ‘unclassified' genus. For the second and third possibilities, ‘unknown' would be the correct classification for such a sequence. When searching for a 100% match for the five most abundant ASVs produced with each of DADA2 in R and DADA2 with QIIME2, in all cases, the sequence was not present in SILVA, GTDB or Greengenes2 but was grouped with an unclassified genus in NCBI (Table S1). Therefore, genus-level classification with Nanopore may be due to alignment with nucleotides outside of the V3–V4 region, or their presence in the NCBI database. Notably, only a small percentage of Illumina ASVs could be completely mapped to Nanopore sequences (Table S3), though a more substantial portion mapped with 95% identity. This mismatch may represent primer bias or errors in Nanopore sequencing. As Nanopore sequencing becomes more common, approaches to assignment should clarify the genetic sequence of each NTU to allow determination of its presence in a database.

## 5. Conclusions

This study utilised widely used sequencing platforms, bioinformatic approaches and taxonomy databases to compare the accuracy of bacterial taxonomy assignments with a mock community of known composition, as well as the alpha diversity of a real dataset. In our study, sequencing with Nanopore followed by processing through NanoFilt and either EPI2ME or Emu performed well at identifying known genera in a mock community, with all profiles produced from Nanopore reads having more similarity to the mock community than any profiles produced from Illumina reads. Within Illumina-sequenced samples, we identified the highest accuracy with a mock community using DADA2 in R and SILVA, while with Nanopore sequences, the best accuracy was with Emu and SILVA. However, some species were less likely to be correctly identified in the mock community, particularly *Salmonella*, across multiple technique combinations. This prevents assurance that the genus-level assignments from Nanopore reads are to the correct bacterial genera, as these bacteria may be more poorly characterised or may be misassigned due to primer bias, bioinformatic approach bias or taxonomy database bias.

Furthermore, these results highlight the dangers of comparing results across studies. Analysis of identical samples with different bioinformatic approaches, and particularly with different taxonomy databases, yielded widely varying relative abundances in some cases and entirely different assignments in others. Whenever possible, we recommend that comparisons be drawn from research conducted with a standard methodology, as differences in each step of the process from sample collection to taxonomy assignment can yield different results [65]. Based on our findings, we conclude that Nanopore sequencing with the 16S rRNA gene Barcoding Kit SQK-RAB204 presents microbiome data comparable to those obtained via Illumina sequencing, both for mock communities and microbiome samples, and may help provide genus- and species-level identification for sequences which cannot be assigned using Illumina. For datasets where genus- or species-level bacterial taxonomy is required for analysis, full-length 16S rRNA gene sequencing with Nanopore presents a viable and promising option. However, all researchers investigating microbial communities should use caution when extrapolating across approaches and consider their choice of taxonomy database, sequencing platform and bioinformatic approach carefully, particularly when examining unknown communities.

## Figures and Tables

**Table 1:** Jaccard distances of the community profile reported by each technique combination as compared to either the mock community sequenced on Illumina or the mock community sequenced on Nanopore. Lower values indicate greater similarity to the mock community. Jaccard distance measures the difference between two sets, with the extreme case that two sets are equal (in this case, the Jaccard value will be 0). This accounts for the number of observations in both sets divided by the number in either set and is represented by the equation (*A* + *B* − 2∗*J*)/(*A* + *B* − *J*), where *J* = sum(*x*∗*y*), *A* = sum(*x*^2) and *B* = sum(*y*^2).

	DADA2 in R/SILVA	DADA2 in R/NCBI	DADA2 in R/GTDB	DADA2 with QIIME2/SILVA	DADA2 with QIIME2/NCBI	DADA2 with QIIME2/Greengenes2
Mock Illumina community	0.685	0.698	0.889	0.751	0.796	0.860

	EPI2ME/SILVA	EPI2ME/NCBI	Emu/SILVA	Emu/NCBI		
Mock Nanopore community	0.500	0.400	0.386	0.409		

## Data Availability

All Illumina sequence data is available at PRJNA1008362. All Nanopore sequence data is available at PRJNA1082425. Detailed workflows for all bioinformatic approaches and processing in R are included in the Supporting Information.

## Supporting Information

Supporting Information 1 Hoffbeck Benchmarking Supplement.pdf contains supplemental figures and tables which are referenced in the text (two figures and three tables).

Supporting Information 2 Detailed pipeline supplemental.pdf contains the bioinformatic code used to process and analyse the presented data.
