# Supplementary material for: Benchmarking 16S rRNA Gene-Based Approaches to Bacterial Taxonomy Assignment Based on Amplicon Sequencing With Illumina and Oxford Nanopore
Source: Int J Microbiol. 2025 Aug 13;2025:7563096. doi: 10.1155/ijm/7563096 (PMC12367389; doi:10.1155/ijm/7563096)
Supplement: Supporting Information 1 — Hoffbeck Benchmarking Supplement.pdf contains supplemental figures and tables which are referenced in the text (two figures and three tables). [file 7563096.f1.docx]

Supplemental Figure 1. Rarefaction curves for ASVs and “pseudo-ASVs” derived from Illumina reads with DADA2 in R and in QIIME2 (row 1) and Nanopore reads with Emu and EPI2ME (row 2).

Supplemental Table 1. Presence and character of top five most abundant ASVs generated each with DADA2 in R and with QIIME2 in all utilised databases.

| DADA2 R | SILVA | GTDB | NCBI | QIIME2 DADA2 | SILVA | Greengenes | NCBI |
| --- | --- | --- | --- | --- | --- | --- | --- |
| ASV1 | LCA "none" | Not in database | Uncultured neighbour found | ASV1 | LCA "none" | Not in database | Uncultured neighbour found |
| ASV2 | LCA "none" | Not in database | Uncultured neighbour found | ASV2 | LCA "none" | Not in database | Uncultured neighbour found |
| ASV3 | LCA "none" | Not in database | Uncultured neighbour found | ASV3 | LCA "none" | Not in database | Uncultured neighbour found |
| ASV4 | LCA "none" | Not in database | Uncultured neighbour found | ASV4 | LCA "none" | Not in database | Uncultured neighbour found |
| ASV5 | LCA "none" | Not in database | Uncultured neighbour found | ASV5 | LCA "none" | Not in database | Uncultured neighbour found |

Supplemental Table 2. The number of sequence reads in initial files, after filtering and trimming, and the final number of processed ASVs or NTUs for each technique combination.

|  | Initial reads | Reads after trimming and filtering | ASVs/NTUs |
| --- | --- | --- | --- |
| **Illumina** |  |  |  |
| DADA2 in R | 30798604 | 30346948 | 40137 |
| DADA2 with QIIME2 | 30798604 | 30346948 | 27815 |
| **Nanopore** |  |  |  |
| EPI2ME NCBI | 16167135 | 15844619 | 3025 |
| EPI2ME SILVA | 16167135 | 15844619 | 2213 |
| Emu NCBI | 16167135 | 15844619 | 5276 |
| Emu SILVA | 16167135 | 15844619 | 2615 |

Supplemental Table 3. Illumina ASVs produced with DADA2 in R and with QIIME2 mapped to Nanopore reads.

|  | Number of reads | Percent of reads |
| --- | --- | --- |
| **100% match** |  |  |
| DADA2 in R | 240 | 0.6% |
| DADA2 with QIIME2 | 73 | 0.3% |
| **95% match** |  |  |
| DADA2 in R | 31608 | 78.8% |
| DADA2 with QIIME2 | 21362 | 76.8% |


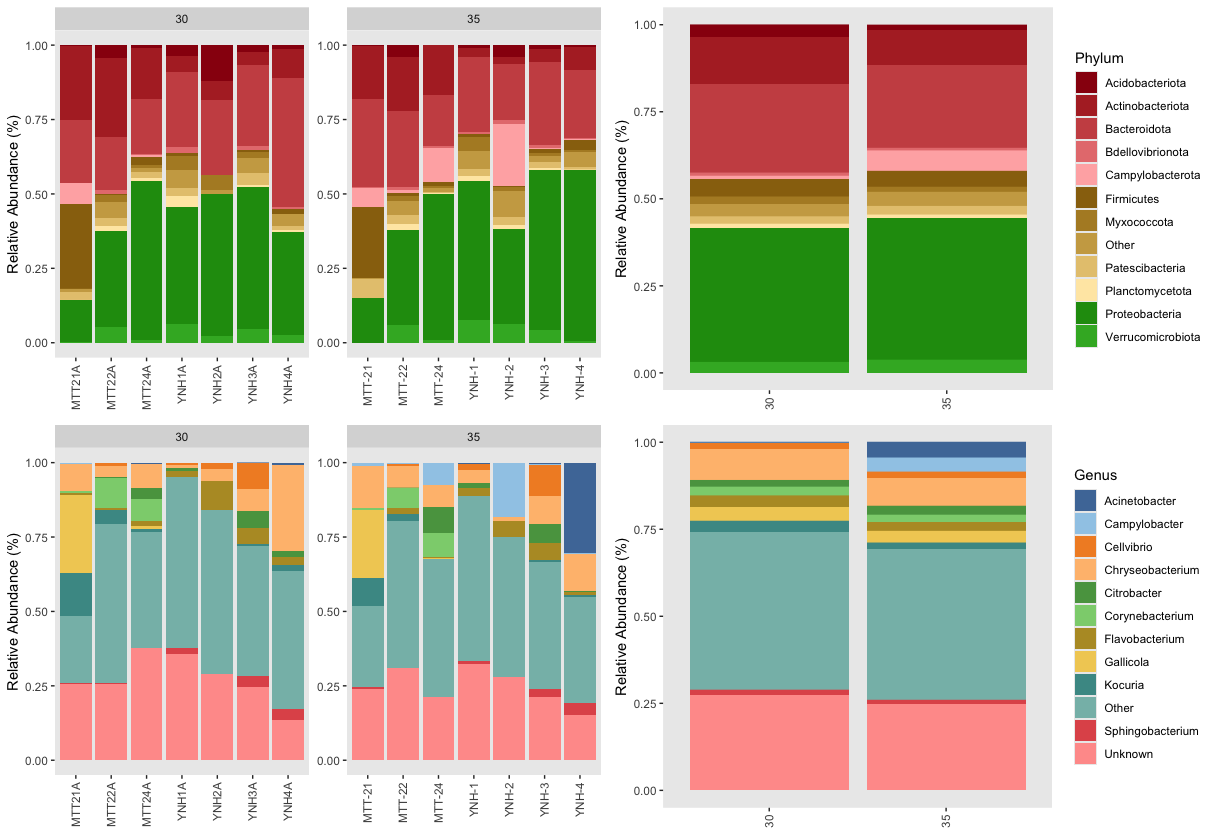


Supplemental Figure 2. Taxonomy comparison for identical samples sequenced with Illumina after amplifying for 30 cycles or 35 cycles. Bacterial diversity was not statistically significantly different between these groups when compared with PERMANOVA (p > 0.05).
