# Supplementary material for: Benchmarking 16S rRNA Gene-Based Approaches to Bacterial Taxonomy Assignment Based on Amplicon Sequencing With Illumina and Oxford Nanopore
Source: Int J Microbiol. 2025 Aug 13;2025:7563096. doi: 10.1155/ijm/7563096 (PMC12367389; doi:10.1155/ijm/7563096)
Supplement: Supporting Information 2 — Detailed pipeline supplemental.pdf contains the bioinformatic code used to process and analyse the presented data. [file 7563096.f2.pdf]

Pipeline for processing tuatara microbiome samples with 10 technique combinations

Illumina sequence reads

Trimmomatic (version 0.39) to filter low-quality reads and trim adaptors

```
trimmomatic PE -threads 4 ${FORWARD} ${REVERSE} \

adapt_trim/${NAME}.fastq.gz adapt_untrim/${NAME}_untrim.fastq.gz \

adapt_trim/${NAME2}.fastq.gz adapt_untrim/${NAME2}_untrim.fastq.gz \

ILLUMINACLIP:NexteraPE-PE.fa:2:40:15
```

DADA2 in R (version 4.2.1)

```
# load libraries

library(dada2); packageVersion("dada2")

library(DECIPHER); packageVersion("DECIPHER")


path <- "/nesi/project/uoa03068/Cam/Latitudinal/adapt_trim"


# forward and reverse fastq filenames have format: SAMPLENAME_R1_001.fastq and SAMPLENAME_R2_001.fastq

fnFs <- sort(list.files(path, pattern="_L001_R1_001.fastq", full.names = TRUE))

fnRs <- sort(list.files(path, pattern="_L001_R2_001.fastq", full.names = TRUE))


# extract sample names

sample.names <- sapply(strsplit(basename(fnFs), "_"), `[` , 2)


# get sample of quality

plotQualityProfile(fnFs[1:4])
```

```
plotQualityProfile(fnRs[1:4])

# place filtered files in filtered/ subdirectory
filtFs <- file.path(path, "filtered", paste0(sample.names, "_F_filt.fastq.gz"))
filtRs <- file.path(path, "filtered", paste0(sample.names, "_R_filt.fastq.gz"))
names(filtFs) <- sample.names
names(filtRs) <- sample.names

# filter and trim
out <- filterAndTrim(fnFs, filtFs, fnRs, filtRs, truncLen=c(280,200),
              maxN=0, maxEE=c(2,2), truncQ=2, rm.phix=TRUE)

# learn errors
errF <- learnErrors(filtFs, multithread=TRUE)
errR <- learnErrors(filtRs, multithread=TRUE)

# sample inference
dadaFs <- dada(filtFs, err=errF, multithread=TRUE)
dadaRs <- dada(filtRs, err=errR, multithread=TRUE)

# merge paired reads
mergers <- mergePairs(dadaFs, filtFs, dadaRs, filtRs, verbose=TRUE)

# construct sequence table
seqtab <- makeSequenceTable(mergers)
```

```
# remove chimeras

seqtab.nochim <- removeBimeraDenovo(seqtab, method="consensus", multithread=TRUE, verbose=TRUE)


# track reads through the pipeline

getN <- function(x) sum(getUniques(x))

track <- cbind(out, sapply(dadaFs, getN), sapply(dadaRs, getN), sapply(mergers, getN), rowSums(seqtab.nochim))


# If processing a single sample, remove the sapply calls: e.g. replace sapply(dadaFs, getN) with getN(dadaFs)

colnames(track) <- c("input", "filtered", "denoisedF", "denoisedR", "merged", "nonchim")

rownames(track) <- sample.names


# assign taxonomy

load("SILVA_SSU_r138_2019.RData")

ids <- assignTaxonomy(seqtab.nochim, trainingSet, multithread=TRUE)

ranks <- c("Domain", "Phylum", "Class", "Order", "Family", "Genus", "Species")

# Convert the output object of class "Taxa" to a matrix

taxid <- t(sapply(ids, function(x) {

    m <- match(ranks, x$rank)

    taxa <- x$taxon[m]

    taxa[startsWith(taxa, "unclassified_")] <- NA

    taxa}))

colnames(taxid) <- ranks; rownames(taxid) <- getSequences(seqtab.nochim)


# giving our seq headers more manageable names (ASV_1, ASV_2...)
```

```
asv_seqs <- colnames(seqtab.nochim)

asv_headers <- vector(dim(seqtab.nochim)[2], mode="character")


for (i in 1:dim(seqtab.nochim)[2]) {

  asv_headers[i] <- paste(">ASV", i, sep="_")}


# making and writing out a fasta of our final ASV seqs:

asv_fasta <- c(rbind(asv_headers, asv_seqs))

write(asv_fasta, "ASVs_dada2_silva.fa")


# count table:

asv_tab <- t(seqtab.nochim)

row.names(asv_tab) <- sub(">", "", asv_headers)

write.table(asv_tab, "ASVs_counts_dada_silva.tsv", sep="\t", quote=F, col.names=NA)


# tax table

write.table(taxid, "ASVs_taxonomy_dada_silva.tsv", sep = "\t", quote=F, col.names=NA)
```

QIIME2 (version 2023.5) with DADA

```
# load metadata table
qiime metadata tabulate \
  --m-input-file metadata_filt.txt --o-visualization metadata-summ-1.qzv

# read in demultiplexed samples
qiime tools import \
  --type 'SampleData[PairedEndSequencesWithQuality]' \
  --input-format CasavaOneEightSingleLanePerSampleDirFmt \
  --input-path files  --output-path demultiplexed-sequences.qza

qiime demux summarize \
  --i-data demultiplexed-sequences.qza --o-visualization demultiplexed-sequences-summ.qzv

# denoise in dada2
qiime dada2 denoise-paired \
  --i-demultiplexed-seqs demultiplexed-sequences.qza \
  --p-trunc-len-f 280 --p-trunc-len-r 200 \
  --o-representative-sequences asv-sequences-0.qza \
  --o-table feature-table-0.qza --o-denoising-stats dada2-stats.qza

# assign taxonomy
qiime feature-classifier classify-sklearn \
  --i-classifier silva138.qza --i-reads asv-sequences-0.qza \
  --o-classification taxonomy.qza
  qiime metadata tabulate \
```

```
  --m-input-file taxonomy.qza \
  --o-visualization taxonomy.qzv

# filter chloroplasts, mitichondria
qiime taxa filter-table \
  --i-table feature-table-0.qza \
  --i-taxonomy taxonomy.qza \
  --p-mode contains \
  --p-include p__ \
  --p-exclude 'Chloroplast,Mitochondria' \
  --o-filtered-table filtered-table-1.qza

# extract ASVs as fasta file
qiime tools export \
   --input-path asv-sequences-0.qza \
   --output-path seqs/

qiime feature-table summarize
   --i filtered-table-1.qza

qiime feature-table summarize
   --i taxonomy.qza
```

NOTE: The above shows processing with SILVA-138. DADA2 and QIIME2 were both again run with the same parameters, with the DADA2 taxonomy database changed to GTDB and the QIIME2 database changed to Greengenes2.

Further taxonomy assigned using command line BLAST (version 2.1.6)

srun blastn -query dna-sequences-dada.fa -db nt -out ASVs_dada_nt.txt -max_target_seqs 5 -num_threads 24 -evalue 1E-10 -outfmt "6 qseqid sacc salltitles pident length evalue sskingdoms ssphylums sscinames"

srun blastn -query dna-sequences-qiime-silva.fa -db nt -out ASVs_qiime_nt.txt -max_target_seqs 5 -num_threads 24 -evalue 1E-10 -outfmt "6 qseqid sacc salltitles pident length evalue sskingdoms ssphylums sscinames"

Nanopore sequence reads

Nanofilt (version 2.6.0) to trim reads and filter by quality

```
for i in {*.fastq}; do
	NanoFilt -q 10 -l 1500 barcode${i}_SupHigh_calls.fastq > barcode${i}_trimmed.fastq
done
```

EPI2ME (via Nextflow version 23.10.0)

```
nextflow run epi2me-labs/wf-16s -profile singularity --fastq 'files_for_epi2me' --classifier kraken2 --watch_path --threads 24 --database_set SILVA_138_1
```

Emu (version 3.4.5)

```
export EMU_PREBUILT_DB='silva'

pip install osfclient

export EMU_DATABASE_DIR=./

osf -p 56uf7 fetch osfstorage/emu-prebuilt/${EMU_PREBUILT_DB}.tar

tar -xvf ${EMU_PREBUILT_DB}.tar

emu abundance trimmed_files/${samples}_trimmed.fastq --db ${EMU_PREBUILT_DB} --keep-counts --output-dir ./emu_silva

emu collapse-taxonomy emu_silva tax_id --counts
```

NOTE: The above shows processing with SILVA-138. EPI2ME and Emu were both again run with the same parameters using the Emu command export EMU_PREBUILT_DB='rdp' **and the EPI2ME command** ncbi_16s_18s_28s_ITS **to assign taxonomy using the NCBI database.**

Further processing in R: once all ten pipelines above produced count tables and taxonomy tables, they were processed in R (version 4.2.1) and merged into a single phyloseq object

```
# clear workspace

rm(list = ls())


# load libraries

library(tidyverse); library(phyloseq); library(microbiome); library(cowplot); library(ggpubr);
library(MiscMetabar)


# load in data

sample_info_mock <- read.table("mock_sample_info.txt", quote = "", header=T,
row.names=1, check.names=F, sep="\t")

count_mock <- read.table("mock_count_table.tsv", header=T, row.names=1,
check.names=F, sep="\t")

tax_mock <- as.matrix(read.table("mock_tax_table.tsv", header=T,  row.names=1,
check.names=F, sep="\t"))


sample_dada_silva <- read.table("dada_silva_info.txt", quote = "", header=T, row.names=1,

                          check.names=F, sep="\t")

count_dada_silva <- read.table("ASVs_counts_dada_silva.tsv", header=T, row.names=1,

                    check.names=F, sep="\t")

tax_dada_silva <- as.matrix(read.table("ASVs_taxonomy_dada_silva.tsv", header=T,

                          row.names=1, check.names=F, sep="\t"))


sample_dada_gtdb <- read.table("dada_gtdb_info.txt", quote = "", header=T, row.names=1,

                          check.names=F, sep="\t")

count_dada_gtdb <- read.table("ASVs_counts_dada_gtdb.tsv", header=T, row.names=1,

                          check.names=F, sep="\t")
```

```
tax_dada_gtdb <- as.matrix(read.table("ASVs_taxonomy_dada_gtdb.tsv", header=T,
                          row.names=1, check.names=F, sep="\t"))

sample_dada_ncbi <- read.table("dada_ncbi_info.txt", quote = "", header=T, row.names=1,
                     check.names=F, sep="\t")
count_dada_ncbi <- read.table("ncbi_dada_count.tsv", header=T, row.names=1,
                    check.names=F, sep="\t")
tax_dada_ncbi <- as.matrix(read.table("ncbi_dada_taxonomy.tsv", header=T,
                          row.names=1, check.names=F, sep="\t"))

sample_qiime_ncbi <- read.table("qiime_ncbi_info.txt", quote = "", header=T, row.names=1,
                     check.names=F, sep="\t")
count_qiime_ncbi <- read.table("ncbi_qiime_count.tsv", header=T, row.names=1,
                    check.names=F, sep="\t")
tax_qiime_ncbi <- as.matrix(read.table("ncbi_qiime_taxonomy.tsv", header=T,
                          row.names=1, check.names=F, sep="\t"))

sample_qiime_silva <- read.table("qiime_silva_info.txt", quote = "", header=T, row.names=1,
                     check.names=F, sep="\t")
count_qiime_silva <- read.table("qiime-silva-counts.tsv", header=T, row.names=1,
                     check.names=F, sep="\t")
tax_qiime_silva <- as.matrix(read.table("qiime-silva-taxonomy.tsv", header=T,
                          row.names=1, check.names=F, sep="\t"))

sample_qiime_greengenes <- read.table("qiime_greengenes_info.txt", quote = "", header=T,
row.names=1, check.names=F, sep="\t")
```

```
count_qiime_greengenes <- read.table("count-table-greengenes2.tsv", header=T,
row.names=1, check.names=F, sep="\t")

tax_qiime_greengenes <- as.matrix(read.table("taxonomy-greengenes2.tsv", header=T,

                              row.names=1, check.names=F, sep="\t"))


sample_epi2me_silva <- read.table("epi2me_silva_info.txt", quote = "", header=T,
row.names=1, check.names=F, sep="\t")

count_epi2me_silva <- read.table("epi2me_silva_count.tsv", header=T, row.names=1,

                        check.names=F, sep="\t")

tax_epi2me_silva <- as.matrix(read.table("epi2me_silva_tax.txt", header=T,

                              row.names=1, check.names=F, sep="\t"))


sample_epi2me_blast <- read.table("epi2me_ncbi_info.txt", quote = "", header=T,
row.names=1, check.names=F, sep="\t")

count_epi2me_blast <- read.table("epi2me_blast_count.tsv", header=T, row.names=1,

                        check.names=F, sep="\t")

tax_epi2me_blast <- as.matrix(read.table("epi2me_blast_tax.txt", header=T,

                              row.names=1, check.names=F, sep="\t"))


sample_emu_silva <- read.table("emu_silva_info.txt", quote = "", header=T, row.names=1,

                         check.names=F, sep="\t")

count_emu_silva <- read.table("emu_silva_count.tsv", header=T, row.names=1,

                      check.names=F, sep="\t")

tax_emu_silva <- as.matrix(read.table("emu_silva_tax.txt", header=T,

                             row.names=1, check.names=F, sep="\t"))


sample_emu_blast <- read.table("emu_ncbi_info.txt", quote = "", header=T, row.names=1,
```

```
                check.names=F, sep="\t")
count_emu_blast <- read.table("emu_blast_count.txt", header=T, row.names=1,
              check.names=F, sep="\t")
tax_emu_blast <- as.matrix(read.table("emu_blast_tax.txt", header=T,
                 row.names=1, check.names=F, sep="\t"))

# make phyloseq objects
count_tab_mock <- otu_table(count_mock, taxa_are_rows=T)
tax_tab_mock <- tax_table(tax_mock)
sample_info_mock <- sample_data(sample_info_mock)
mock_physeq <- phyloseq(count_tab_mock, tax_tab_mock, sample_info_mock)

count_tab_ds <- otu_table(count_dada_silva, taxa_are_rows=T)
tax_tab_ds <- tax_table(tax_dada_silva)
sample_info_dada_silva <- sample_data(sample_dada_silva)
dada_silva_physeq <- phyloseq(count_tab_ds, tax_tab_ds, sample_info_dada_silva)

count_tab_dg <- otu_table(count_dada_gtdb, taxa_are_rows=T)
tax_tab_dg <- tax_table(tax_dada_gtdb)
sample_info_dada_gtdb <- sample_data(sample_dada_gtdb)
dada_gtdb_physeq <- phyloseq(count_tab_dg, tax_tab_dg, sample_info_dada_gtdb)

count_tab_dn <- otu_table(count_dada_ncbi, taxa_are_rows=T)
tax_tab_dn <- tax_table(tax_dada_ncbi)
sample_info_dada_ncbi <- sample_data(sample_dada_ncbi)
dada_ncbi_physeq <- phyloseq(count_tab_dn, tax_tab_dn, sample_info_dada_ncbi)
```

```
count_tab_qs <- otu_table(count_qiime_silva, taxa_are_rows=T)
tax_tab_qs <- tax_table(tax_qiime_silva)
sample_info_qiime_silva <- sample_data(sample_qiime_silva)
qiime_silva_physeq <- phyloseq(count_tab_qs, tax_tab_qs, sample_info_qiime_silva)

count_tab_qg <- otu_table(count_qiime_greengenes, taxa_are_rows=T)
tax_tab_qg <- tax_table(tax_qiime_greengenes)
sample_info_qiime_gg <- sample_data(sample_qiime_greengenes)
qiime_greengenes_physeq <- phyloseq(count_tab_qg, tax_tab_qg, sample_info_qiime_gg)

count_tab_qn <- otu_table(count_qiime_ncbi, taxa_are_rows=T)
tax_tab_qn <- tax_table(tax_qiime_ncbi)
sample_info_qiime_ncbi <- sample_data(sample_qiime_ncbi)
qiime_ncbi_physeq <- phyloseq(count_tab_qn, tax_tab_qn, sample_info_qiime_ncbi)

count_tab_es <- otu_table(count_epi2me_silva, taxa_are_rows=T)
tax_tab_es <- tax_table(tax_epi2me_silva)
sample_info_epi2me_silva <- sample_data(sample_epi2me_silva)
epi2me_silva_physeq <- phyloseq(count_tab_es, tax_tab_es, sample_info_epi2me_silva)

count_tab_eb <- otu_table(count_epi2me_blast, taxa_are_rows=T)
tax_tab_eb <- tax_table(tax_epi2me_blast)
sample_info_epi2me_ncbi <- sample_data(sample_epi2me_blast)
epi2me_blast_physeq <- phyloseq(count_tab_eb, tax_tab_eb, sample_info_epi2me_ncbi)
```

```
count_tab_ems <- otu_table(count_emu_silva, taxa_are_rows=T)

tax_tab_ems <- tax_table(tax_emu_silva)

sample_info_emu_silva <- sample_data(sample_emu_silva)

emu_silva_physeq <- phyloseq(count_tab_ems, tax_tab_ems, sample_info_emu_silva)


count_tab_emb <- otu_table(count_emu_blast, taxa_are_rows=T)

tax_tab_emb <- tax_table(tax_emu_blast)

sample_info_emu_blast <- sample_data(sample_emu_blast)

emu_blast_physeq <- phyloseq(count_tab_emb, tax_tab_emb, sample_info_emu_blast)


# tax_glom on genus and merge

ds_glom <- tax_glom(dada_silva_physeq,taxrank="Genus",bad_empty = c(NA, "", " ",
"\t"),NArm=F)

dg_glom <- tax_glom(dada_gtdb_physeq,taxrank="Genus",bad_empty = c(NA, "", " ",
"\t"),NArm=F)

dn_glom <- tax_glom(dada_ncbi_physeq,taxrank="Genus",bad_empty = c(NA, "", " ",
"\t"),NArm=F)

qs_glom <- tax_glom(qiime_silva_physeq,taxrank="Genus",bad_empty = c(NA, "", " ",
"\t"),NArm=F)

qg_glom <- tax_glom(qiime_greengenes_physeq,taxrank="Genus",bad_empty = c(NA, "", " ",
"\t"),NArm=F)

qn_glom <- tax_glom(qiime_ncbi_physeq,taxrank="Genus",bad_empty = c(NA, "", " ",
"\t"),NArm=F)

es_glom <- tax_glom(epi2me_silva_physeq,taxrank="Genus",bad_empty = c(NA, "", " ",
"\t"),NArm=F)

en_glom <- tax_glom(epi2me_blast_physeq,taxrank="Genus",bad_empty = c(NA, "", " ",
"\t"),NArm=F)

ems_glom <- tax_glom(emu_silva_physeq,taxrank="Genus",bad_empty = c(NA, "", " ",
"\t"),NArm=F)
```

```
emn_glom <- tax_glom(emu_blast_physeq,taxrank="Genus",bad_empty = c(NA, "", " ",
"\t"),NArm=F)

glom_phy <- merge_phyloseq(ds_glom,dg_glom,dn_glom,qs_glom,qg_glom,qn_glom,
                    es_glom,en_glom,ems_glom,emn_glom)

glom_phy_glom <- tax_glom(glom_phy,taxrank="Genus",bad_empty = c(NA, "", " ",
"\t"),NArm=F)

final_phy <- clean_pq(glom_phy_glom,remove_empty_samples =  TRUE,
remove_empty_taxa = TRUE)

r_count <- as.data.frame(as(otu_table(final_phy), "matrix"))
r_sample <- as.data.frame(as(sample_data(final_phy), "matrix"))

ord <- ordinate(final_phy, method = "NMDS", distance = "bray", trymax = 50, autotransform
= TRUE)

permanova <- adonis2(t(r_count) ~ Pipeline*Database*Read_type,
            data = r_sample, permutations=999, method = "bray", by="margin")
```
